# Polynuclear Aromatic Hydrocarbons in Port Valdez Shrimp and Sediment

**DOI:** 10.1007/s00244-016-0279-3

**Published:** 2016-03-31

**Authors:** Mark G. Carls, Larry Holland, Erik Pihl, Marilyn A. Zaleski, John Moran, Stanley D. Rice

**Affiliations:** NOAA/NMFS, Auke Bay Laboratories, 17109 Point Lena Loop Road, Juneau, AK 99801 USA

## Abstract

Polynuclear aromatic hydrocarbons (PAHs) from oil were present in some shrimp from Port Valdez, site of a ballast water treatment facility at the Alyeska Alaska Marine Terminal (AMT). Low-level petrogenic PAH concentrations were generally restricted to shrimp eggs in the vicinity of the AMT and extended along the southern shore of Port Valdez to Anderson Bay. Eggs had greater lipid content than other tissues and thus were the most vulnerable biological compartment to hydrocarbon accumulation. Petrogenic hydrocarbons were not observed in shrimp muscle and cephalothoraxes; thus, these tissues do not pose a human health risk. Risk for children older than age 2 years and adults consuming eggs also was low except for two unusual samples (of 32), collected about 17 km west of the treatment facility. In general, PAH loads were consistent with local time series data in other species. We infer that the accumulation mechanism was dissolved uptake from water, consistent with passive sampler observations completed more than a decade earlier. Hydrocarbon levels in the majority of samples were below toxic thresholds. Total PAH accumulation was substantially greater in some pink shrimp than in other species, thus differences in habitat utilization (muddy vs. rocky substrate) are potentially important.

**Electronic supplementary material:**

The online version of this article (doi:10.1007/s00244-016-0279-3) contains supplementary material, which is available to authorized users.

This study explores the possibility that hydrocarbon discharge from oil tanker operations in Port Valdez, Alaska, may affect the reproductive capacity of indigenous shrimp or pose a health risk to human consumers of them. The goal was to measure polynuclear aromatic hydrocarbon (PAH) loads in eggs, edible muscle tissue, and cephalothoraxes of shrimp to determine if the source of observed PAHs was consistent with oil, and if observed, concentrations pose a health risk to consumers or to the reproductive capacity of the shrimp. Division by tissue type helped distinguish water-borne and ingestion exposure routes and compare differences in external and internal sequestration. Sediment was collected when and where shrimp were collected to assess the exposure potential for shrimp utilizing this habitat and to determine hydrocarbon sources.

Hydrocarbon release from oil tanker operations in Port Valdez, Alaska, has the potential to contaminate organisms living in the area. The Port Valdez, Alaska, ecosystem receives about 10^7^ L/day of oil-contaminated ballast water from a treatment facility at the southern terminus of the trans-Alaska oil pipeline (Payne et al. 2005). Petrogenic hydrocarbon inputs from the Alyeska Marine Terminal (AMT) and tanker operations have been declining in bay mussels (*Mytilus trossulus*) and sediments in recent years (Payne et al. 2013) but remain detectable and continue to raise concerns among people who harvest food from this area. Discharge has likely declined due to a combination of reduced ballast-water-treatment-facility discharge volumes from decreased North Slope oil production, the transition to double-hulled tankers with segregated ballast tanks, and improved treatment efficiency at removing particulate/oil-phase polycyclic aromatic hydrocarbons (PAHs) (Payne et al. 2013). However, the severity of winter weather typically encountered between Valdez and other west coast ports requires more ballast than can be carried in segregated tanks, thus the need to process oily ballast water continues (PWSRCAC 2014).

Shrimp are among the organisms in Port Valdez potentially contaminated by PAHs, the toxic compounds of concern in crude oil. Shrimp are harvested in Prince William Sound using either pots or trawl gear. The pot fishery is directed towards spot shrimp (*Pandalus platyceros*) and to a more limited extent, coonstripe (*P. hypsinotus*) shrimp. The trawl fishery targets pink shrimp (*P. borealis*). Port Valdez is included in the “Inside” management district and is open for the trawl fishery but not for the pot fishery, although personal-use pots are allowed (Wessel et al. 2012). The pot and trawl fisheries began in the 1960s and 1970s, respectively, and both harvests peaked in the 1980s followed by reduced landings of >60–90 % by 1989 (Armstrong et al. 1995; Wessel et al. 2012). Before the *Exxon Valdez* oil spill, decreases in catch were seen in pink, coonstripe, sidestripe (*P. dispar*), and humpy shrimp (*P. goniurus*) associated with both fishing pressure (Kimker et al. 1996; Trowbridge 1992) and increased water temperature (Anderson and Piatt 1999). Catches in 1989 also were greatly depressed due to the Exxon oil spill limiting vessel access to fishing grounds (Wessel et al. 2012), although both fisheries persisted through the next decade. The pot fishery closed in 2000 but reopened 2010 with a season-limited fishery to avoid gravid females (Wessel et al. 2012).

Habitat utilization varies among the three *Pandalid* species chosen for study. Pink and coonstripe shrimp are found on smooth muddy to sandy bottoms, whereas spot shrimp occur in steep, rocky areas (Butler 1964). All three species are protandrous hermaphrodites and first mature as males before undergoing a sex transformation into functional females at approximately 4.5, 3, and 6 years, respectively (Armstrong et al. 1995; Orensanz et al. 1998). Variability in the mean age and size at sex change can vary with both population density and geography (Anderson 1991; Armstrong et al. 1995). The ovigerous period of mature female shrimp varies among species, ranging from 7 to 8, 6 to 11, and 4 to 5.5 months in pink, coonstripe, and spot shrimp, respectively (Orensanz et al. 1998; Shumway et al. 1985).

We hypothesized that shrimp species with the most contact with soft sediment are the most likely to become contaminated, because previous study has demonstrated that sediment is a compartment that accumulates oil residue in Port Valdez. We further hypothesized that shrimp eggs would be the most likely tissue to accumulate PAHs if transfer involved constituents dissolved in water. The relatively high lipid content of egg tissue and their external position on shrimp should render them vulnerable to PAH accumulation similar to lipid-rich copepods and passive samplers (Carls et al. 2004; Salazar et al. 2002). Dissolved PAHs also have been detected in bay mussels in Port Valdez (Payne et al. 2005), although these filter-feeding organisms can accumulate oil droplets when the water column is not stratified (Payne et al. 2005).

## Sample Area

**Fig. 1:**
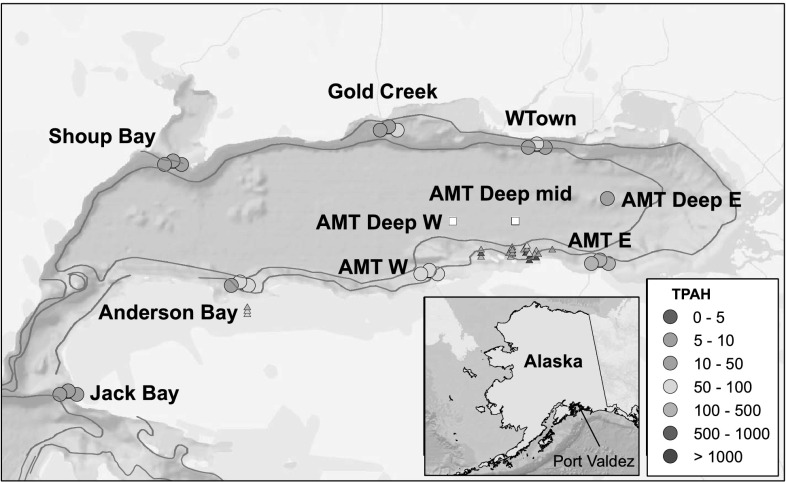
Port Valdez, Alaska, sample area. Total PAH concentrations are illustrated for coonstripe shrimp eggs (ng/g dry wt; *circles*) and passive samplers (ng/g device; *triangles*). The latter are from a previous study and are included as discussion in this paper. *Small white squares* are sample locations without coonstripe shrimp (Color figure online)

Port Valdez is a narrow fjord located in the northeast corner of Prince William Sound (PWS), Alaska, and is separated from PWS by a narrow channel and double sill (Fig. 1). The walls of this approximately 100-km^2^ basin drop steeply to a nearly flat bottom approximately 240-m deep; mean depth is approximately 180 m (Colonell 1980). Tidal exchange is large, approximately 5.6 m maximum and 3.0 m mean. Mean annual precipitation is 158 cm per year (Blanchard and Feder 2000a, b). The suspended sediment load is seasonal; high loads coincide with spring melt (Feder and Keiser 1980; Sharma and Burbank 1973). Subsurface flow patterns (15 m below the surface) tend to be irregular, with an ill-defined wind-driven western movement (Muench and Nebert 1973). Water exchange between PWS and Port Valdez is influenced by tidal flushing, seasonal deep water exchange, and weather-driven events. The tidal prism is approximately 1.6 % of the total volume, suggesting the half-life of a conservative (i.e., degradation-resistant) pollutant is approximately 22 days in dry weather (Colonell 1980). Deep water exchange is enhanced during summer and early autumn as surface freshwater outflow is replaced by more saline deeper water from PWS via the Valdez Narrows (Colonell 1980; Muench and Nebert 1973; Sharma and Burbank 1973). Surges of surface water from PWS into Port Valdez and large outflows at depths >50 m are apparently related to weather systems and are believed to be the processes with the greatest influence on deep water exchange (Colonell 1980).

## Methods

Nine sites were sampled February 26–27, 2013 within Port Valdez, Alaska, and one additional site, Jack Bay, was sampled adjacent to the Valdez Narrows (Fig. 1; Table 1). Sites were chosen to allow comparison between reference areas and areas receiving hydrocarbon input as a result of ballast water treatment at the AMT. The sites also were chosen to allow comparison to previous studies (Carls et al. 2006; Payne et al. 2013; Salazar et al. 2002). Jack Bay was chosen as a nearby reference site outside the entrance sill into Port Valdez and distant from ballast water effluent. Some locations are used by local shrimp fishermen (West Town, Gold Creek, Shoup Bay, and Anderson Bay). The AMT West and AMT East sites were relatively shallow sport fishing areas (60–86 m). Pink shrimp were more likely to be present than the other species in the deeper AMT sites (197–237 m). Each site was typically sampled with four 80-cm diameter by 30-cm tall shrimp pots with 2-cm mesh; 1 of 12 pots had approximately 3-cm mesh. No shrimp were captured at AMT West Deep, even though 5 pots were used. Shrimp were separated by species, wrapped in aluminum foil, placed in Ziploc©^1^ bags, and frozen immediately after capture.

**Table 1:** Shrimp sample locations, depth, effort (number of pots), and numbers captured with and without eggs

Location	Coonstripe shrimp		Spot shrimp		Pink shrimp		Depth (m)	*n* Pots
	w/eggs	No eggs	w/eggs	no eggs	w/eggs	No eggs		
Shoup Bay	9		1	4			60	4
Gold Creek	8			6		5	84	3
West Town	3	5	1	5			67	4
AMT East Deep	1		1		9	7	197	4
AMT East		5		5	1		46	4
AMT Mid Deep^a^						many	234	3
AMT West Deep^b^							237	4
AMT West	8			5			87	5
Anderson	4	5		5	2	15	68	4
Jack Bay^c^	4	5		5	1		67	4

^a^Small shrimp

^b^No shrimp

^c^Reference site outside Port Valdez

Benthic sediment was collected with a Van Veen grab in close proximity to shrimp pot placement. The grab preserved sediment structure and the top 2 or 3 mm were sampled with a hydrocarbon-free spoon to yield samples of recent sediment. These were frozen pending analysis.

Shrimp tissues were subdivided into egg, muscle, and cephalothorax samples in the laboratory before further analysis. Shrimp were thawed, weighed to the nearest 0.001 g, measured (carapace length in mm), and dissected within 15 min of thawing. Shrimp were handled with hydrocarbon-free forceps, scalpels, and scissors. Subsampled tissues were placed in hydrocarbon-free jars and frozen pending hydrocarbon extraction.

### Lipid Measurement

Lipids were measured to understand the relative ability of the three tissue types to accumulate hydrocarbons. Analysis among tissues was restricted to coonstripe shrimp, where an adequate number of samples were available for each tissue type (6 ≤ *n* ≤ 8). Additional available spot shrimp eggs (*n* = 1) and pink shrimp eggs (*n* = 2) were measured for an among-species egg comparison. Lipid was extracted from roughly 0.250 g of wet sample homogenate using a 1:1 chloroform methanol solution (Christie 1982). Liquid–liquid extractions were performed using an aqueous solution of 0.88 % potassium chloride (0.88 g/100 ml) equal to one-quarter of the extraction volume. The bottom layer of biphasic solution was collected and reextracted by using a 1:1 methanol deionized water solution at one-quarter of the volume of the recovered extract. The bottom extraction layer was collected and its volume reduced to 1 mL under vacuum using a rotary evaporator. Gravimetric analysis of the lipid extract was used to determine the percent lipid expressed as a percentage of the wet tissue mass.

### Hydrocarbon Measurement

Hydrocarbons were extracted from tissue and sediment with dichloromethane, dried, fractionated, purified, and processed by gas chromatography–flame ionization detection (GC–FID) and gas chromatography-mass spectroscopy (GC–MS). Tissue was spiked with 500 μl of deuterated surrogate recovery standard (Table 2) and then extracted with dichloromethane using a Dionex accelerated solvent extractor (Larsen et al. 2015). Tissue and sediment extracts were dried with sodium sulfate and concentrated to 1 ml in hexane. The extracts were applied to a chromatography column (20 g of 5 % deactivated silica gel over 10 g of 2 % deactivated alumina) and separated into aliphatic and aromatic fractions. The aliphatic compounds were eluted with 50 ml of pentane, and aromatic compounds were eluted with 250 ml of a 1:1 mixture of pentane and dichloromethane. Tissue aromatic fractions were further purified by a high-performance liquid chromatography equipped with size-exclusion columns (22.5 mm × 250 mm, phenogel, 100 angstrom pore size). Both the aliphatic and the aromatic fractions were reduced to 1 ml in hexane, spiked with internal standards (dodecylcyclohexane and hexamethylbenzene, respectively), and stored at −20 °C pending GC analysis.

**Table 2:** Deuterated surrogate polynuclear aromatic hydrocarbon (PAH) standards and concentrations in spike used for tissue and sediment

μg/ml	Surrogate
2.0	Naphthalene-d_8_
2.0	Acenaphthene-d_10_
2.0	Phenanthrene-d_10_
2.0	Chrysene-d_12_
2.0	Perylene-d_12_
2.0	Benzo[*a*]pyrene-d_12_
9.9	*n*-Dodecane-d_26_
9.7	*n*-Hexadecane-d_34_
9.7	*n*-Eicosane-d_42_
9.8	*n*-Tetracosane-d_50_
9.7	*n*-Triacontane-d_62_

Spike volumes were 500 μl for tissue and sediments. The spike solvent was hexane

Aliphatic fractions were analyzed for *n*-alkanes using GC–FID. Analyte concentrations were determined by the internal standard method. Experimentally determined method detection limits were approximately 5 ng/g in tissue and <1 ng/g in sediment. The accuracy of the alkane analyses was ±11 % based on a spiked blank processed with each set of samples and precision expressed as coefficient of variation was usually less than approximately 20 %. Surrogate recoveries averaged 57 and 58 % in tissue and sediment, respectively. Total alkane concentrations were calculated by summing concentrations of individual calibrated alkanes [*n*-C9 through *n*-C36 alkanes plus pristane (PRI) and phytane (PHY)]. The unresolved complex mixture (UCM) concentration was determined from the difference between the total FID response area and resolved peak areas. Concentrations are reported as ng/g dry weight.

Aromatic fractions were analyzed for PAHs by GC–MS. Data were acquired in selected ion monitoring (SIM) mode and concentrations were determined by the internal standard method (Short et al. 1996). Experimentally determined method detection limits were approximately 0.3 ng/g in tissue and 0.04 ng/g in sediment. The accuracy of the PAH analyses was approximately ±15 % based on comparison with National Institute of Standards and Technology values (SRM1944), and precision expressed as coefficient of variation was approximately 30 %, depending on the PAH. Surrogate recoveries averaged 60 and 74 % in tissue and sediment, respectively. Total PAH (TPAH) concentrations were calculated by summing concentrations of individual PAH except perylene, which occurs naturally, was not included in the total. Measured PAHs are listed in Table 3. Concentrations are reported as ng/g dry weight.

**Table 3:** Measured polynuclear aromatic hydrocarbons, their abbreviations, and toxicity equivalent factors with respect to BAP (Nisbet and Lagoy 1992)

Abbreviation	Compound	TEF
N0	Naphthalene	0.001
N1	C-1 naphthalenes	0.001
N2	C-2 naphthalenes	*0.001*
N3	C-3 naphthalenes	*0.001*
N4	C-4 naphthalenes	*0.001*
BPH	Biphenyl	*0.001*
ACN	Acenaphthylene	0.001
ACE	Acenaphthylene	0.001
F0	Fluorene	0.001
F1	C-1 fluorenes	*0.001*
F2	C-2 fluorenes	*0.001*
F3	C-3 fluorenes	*0.001*
F4	C4 fluorenes	*0.001*
D0	Dibenzothiophene	*0.001*
D1	C-1 dibenzothiophenes	*0.001*
D2	C-2 dibenzothiophenes	*0.001*
D3	C-3 dibenzothiophenes	*0.001*
D4	C4 dibenzothiophenes	*0.001*
P0	Phenanthrene	0.001
P1	C-1 phenanthrenes/anthracenes	*0.001*
P2	C-2 phenanthrenes/anthracenes	*0.001*
P3	C-3 phenanthrenes/anthracenes	*0.001*
P4	C-4 phenanthrenes/anthracenes	*0.001*
ANT	Anthracene	0.01
FLU	Fluoranthene	0.001
PYR	Pyrene	0.001
FP1	C-1 fluoranthenes/pyrenes	*0.001*
FP2	C-2 fluoranthenes/pyrenes	*0.001*
FP3	C-3 fluoranthenes/pyrenes	*0.001*
FP4	C-4 fluoranthenes/pyrenes	*0.001*
BAA	Benzo(a)anthracene	0.1
C0	Chrysene	0.01
C1	C-1 chrysenes	*0.01*
C2	C-2 chrysenes	*0.01*
C3	C-3 chrysenes	*0.01*
C4	C-4 chrysenes	*0.01*
BBF	Benzo(b)fluoranthene	0.1
BKF	Benzo(k)fluoranthene	0.1
BEP	Benzo(e)pyrene	*1*
BAP	Benzo(a)pyrene	1
PER	Perylene	0
ICP	Indeno(1,2,3-cd)pyrene	0.1
DBA	Dibenzo(a,h)anthracene	5
BZP	Benzo(ghi)perylene	0.01

Values in italics are estimates for analytes not reported in the literature

Aliphatic fractions of sediments were analyzed for biomarkers by GC–MS (Table 4). The data were acquired in a selected-ion monitoring mode, and concentrations were determined by the internal standard method with response factors (RF) based on two representative compounds: 17α(H),21β(H)-hopane (H30) and 5α(H),14α(H),17α(H)-cholestane. The accuracy of the biomarker analyses was approximately ±15 % based on a spiked blank processed with each set of samples, and precision expressed as coefficient of variation was approximately 20 %, depending on the biomarker. Biomarker concentrations were not corrected for recovery; surrogate recovery averaged 70 % (range 55–76 %).

**Table 4:** Measured biomarkers, abbreviations, and target ions (m/z)

Biomarker: Triterpanes	Target ions
C23 tricyclic terpane	191
C24 tricyclic terpane	191
C25 tricyclic terpane (a)	191
C25 tricyclic terpane (b)	191
C24 tetracyclic terpane	191
C26 tricyclic terpane (a)	191
C26 tricyclic terpane (b)	191
C28 tricyclic terpane (a)	191
C28 tricyclic terpane (b)	191
C29 tricyclic terpane (a)	191
C29 tricyclic terpane (b)	191

Biomarker: hopanes	Target ions
18α(H),21β(H)-22,29,30-trisnorhopane	191
17α(H),21β(H)-22,29,30-trisnorhopane	191
17α(H),18α(H),21β(H)-28,30-bisnorhopane	191
17α(H),21β(H)-25-norhopane	191
17α(H),21β(H)-30-norhopane	191
18α(H),21β(H)-30-norneohopane	191
17α(H),21β(H)-30-norhopane (normoretane)	191
18α(H) and 18β(H)-oleanane	191
17α(H),21β(H)-hopane	191
17α(H)-30-nor-29-homohopane	191
17β(H),21α(H)-hopane (moretane)	191
22S-17α(H),21β(H)-30-homohopane	191
22R-17α(H),21β(H)-30-homohopane	191
Gammacerane	191
22S-17α(H),21β(H)-30,31-bishomohopane	191
22R-17α(H),21β(H)-30,31-bishomohopane	191
22S-17α(H),21β(H)-30,31,32-trishomohopane	191
22R-17α(H),21β(H)-30,31,32-trishomohopane	191
22S-17α(H),21β(H)-30,31,32,33-tetrakishomohopane	191
22R-17α(H),21β(H)-30,31,32,33-tetrakishomohopane	191
22S-17α(H),21β(H)-30,31,32,33,34-pentakishomohopane	191
22R-17α(H),21β(H)-30,31,32,33,34-pentakishomohopane	191

Biomarker: steranes	Target ions
C_22_ 5α(H),14β(H),17β(H)-sterane	217,218
C_27_ 20S-13β(H),17α(H)-diasterane	217,218
C_27_ 20R-13β(H),17α(H)-diasterane	217,218
C_27_ 20S-5α(H),14α(H),17α(H)-cholestane	217,218
C_27_ 20R-5α(H),14β(H),17β(H)-cholestane	217,218
C_27_ 20S-5α(H),14β(H),17β(H)-cholestane	217,218
C_27_ 20R-5α(H),14α(H),17α(H)-cholestane	217,218
C_28_ 20S-5α(H),14α(H),17α(H)-ergostane	217,218
C_28_ 20R-5α(H),14β(H),17β(H)-ergostane	217,218
C_28_ 20S-5α(H),14β(H),17β(H)-ergostane	217,218
C_28_ 20R-5α(H),14α(H),17α(H)-ergostane	217,218
C_29_ 20S-5α(H),14α(H),17α(H)-stigmastane	217,218
C_29_ 20R-5α(H),14β(H),17β(H)-stigmastane	217,218
C_29_ 20S-5α(H),14β(H),17β(H)-stigmastane	217,218
C_29_ 20R-5α(H),14α(H),17α(H)-stigmastane	217,218

### Interpretation and Statistics

Composition of all PAHs in eggs was analyzed by principal components analysis (PCA) to reduce the number of data dimensions, thus providing insight into the presence (or absence) of oil constituents, weathering, and geographic patterns. Concentration data were normalized before analysis (PAH_*i*_/TPAH) to explore composition similarities and differences without complication by variable concentration. PCA was completed with Minitab using correlation matrices, yielding four principal component values for each sample. Sediment data were similarly analyzed.

Composition of PAH was also modeled to characterize source attributes [petrogenic (oil) or pyrogenic] (Carls 2006; Carls et al. 2015); values ranged from −1 (pyrogenic) to +1 (petrogenic). Concentration patterns within six homologous families, naphthalenes (N0–N4), fluorenes (F0–F4), dibenzothiophenes (D0–D4), phenanthrenes (P0–P4), fluoranthene-pyrenes (FL, PY, FP1–FP4), and chrysenes (C0–C4) are examined for petrogenic (rounded hump, peaking in alkylated compounds) or pyrogenic patterns (where parent concentrations are greatest). Scores within any given homologous family range from −1 (pyrogenic) to +1 (petrogenic), and the final score is scaled to range from −1 to +1 by dividing by the number of homologous families contributing to the score.

Analysis of variance (ANOVA) was used to compare various groups followed by pairwise comparison (Holm-Sidak). When assumptions failed, Kruskal–Wallis one-way ANOVA on ranks was used followed by pairwise comparisons (Dunn’s method). Analyses were completed with SigmaPlot software. Median responses are compared to avoid undue bias by high outlier values.

### Risk Assessment for Human Consumption

Cancer risk was estimated using BAP toxic equivalent factors (TEFs; Table 3) (Cai et al. 2012; Kumar et al. 2015; Nisbet and Lagoy 1992; Zhao et al. 2014), because BAP is the only PAH for which a cancer slope factor is available (USEPA 2000). Quantitative PAH risk assessment recognizes that potential potency varies among PAHs, hence observed concentrations (ng/g wet weight) were multiplied by TEF estimates (Nisbet and Lagoy 1992). With few exceptions, TEFs are defined only for the 16 priority PAHs in the literature (Cai et al. 2012). Gaps were filled by using homolog values. For example the TEF for N2, N3, and N4 was set equal to the TEF for N1 [2-methylnaphthalene; (Nisbet and Lagoy 1992)]. The TEF for dibenzothiophenes was set equal to that for phenanthrene, because no literature provided an estimate for these compounds. The TEF for BEP was set equal to that of BAP. Although risk might be underestimated because noncancerous toxicity increases with alkyl substitution (Black et al. 1983; Rice et al. 1977), some authors suggest the addition of side chains has no effect on carcinogenic potency (Bolger et al. 1996).

Potency equivalent concentrations (PECs) of total PAHs were defined as PEC = ∑(TEF_i_ · *C*_i_), where *C*_i_ was the concentration of each PAH (USEPA 2000). All calculations were completed with wet weights to match methods in the literature. Resultant PECs were compared with the value suggested by the USEPA (USEPA 2000) for human fish consumption (0.67 ng/g wet weight for subsistence use, 5.47 ng/g ww for recreational use).

The index of additive lifetime cancer risk (ILCR) was estimated as ILCR = (ED · EF · CR · C · SF · CF)/(BW · AT), where ED is the exposure duration (70 years for adults, 12 years for children), EF is the exposure frequency (365 day/year), CR is the consumption rate (30.5 g/person/day), *C* = *C*_i_ * TEF_i_ (ng/g ww), SF is the oral cancer slope factor for BAP (7.3 mg/kg/day), CF is a conversion factor 10^−6^ mg/ng), BW is body weight (80 kg for adults, 35 kg for children), and AT is the average lifespan for carcinogens (25,550 days for adults, 4380 days for children) (Kumar et al. 2015; Zhao et al. 2014). To further address carcinogenicity and mutagenicity potential in children, an additional EPA method was considered (USEPA 2005). An age-dependent adjustment factor (ADAF) was included in the children’s ILCR model, and ages were subdivided from 1 to 2, 2 to 16, and 16 to adult (ADAF values were 10, 3, and 1, respectively). Subdivision estimates were added together. The mass estimate for toddlers (ages 1–2 years) was 11.2 kg based on U.S. Centers for Disease Control and Prevention growth charts (50th percentile). Risk was considered serious when the ILCR >10^−4^, possible when the ILCR >10^−5^, and acceptable near 10^−6^ and below (CCME 2010a, b; USEPA 2000; Zhao et al. 2014). Health Canada considers an incremental risk of less than 1 in 10^5^ to 10^6^ to be essentially negligible.

## Results

Shrimp were successfully collected from all sites except AMT West Deep; all three species were captured. Egg-bearing coonstripe shrimp were present at most locations; very few spot shrimp had eggs. Pink shrimp were encountered less frequently than other species. Spot shrimp were the largest and pink shrimp were the smallest, consistent with typical species size [Supporting Information (SI) 1]. Egg mass increased with shrimp size (SI 1).

### Tissue

Percent lipid content was significantly greater in coonstripe eggs (8.7 %, range 4.8–10.7) than in cephalothoraxes (2.6 %, range 1.7–3.7) and muscle (1.5 %, range 1.1–1.7; P_Holm-Sidak_ <0.05). Percent lipid in spot shrimp eggs (8.6 %; *n* = 1) and pink shrimp eggs (6.1–8.5; *n* = 2) was within the range observed for coonstripe eggs.

Hydrocarbons varied among tissue and species. The median TPAH concentration was least in muscle and greatest in eggs, although ranges overlapped (Table 5). The total alkane concentration was least in muscle and greatest in cephalothoraxes (Table 5). No UCMs were observed in tissue. Alkanes typically accounted for 85 % of the hydrocarbons detected in eggs, >99 % in cephalothoraxes, and 9 % in muscle. Evidence of oil was discovered in some eggs but not in muscle and cephalothoraxes; PAH composition was petrogenic in those eggs.

**Table 5:** Summary of hydrocarbons and lipids in shrimp tissue: total polynuclear aromatic hydrocarbons (TPAH) and calibrated *n*-alkanes (cAlk)

		TPAH (ng/g dry wt)				cAlk (ng/g dry wt)			Lipid (%)			
		*n*	Median	Min	Max	Median	Min	Max	*n*	Median	Min	Max
Muscle	Coon	22	0.6	0.0	8.5	0	0	144	8	1.5	1.1	1.7
Muscle	Spot	7	2.9	0.0	6.9	0	0	135		–	–	–
Muscle	Pink	12	10.9	0.0	49.4	0	0	230		–	–	–
c-Thorax^a^	Coon	22	4.7	0.0	16.0	6641	931	27,343	7	2.3	1.7	3.7
c-Thorax^a^	Spot	16	11.2	3.6	36.2	12,904	759	44,307		–	–	–
c-Thorax^a^	Pink	13	88.0	14.2	235.7	30,432	3730	124,512		–	–	–
Egg	Coon	22	46.3	5.3	270.4	932	0	22,833	6	9.6	4.8	10.7
Egg	Spot	2	12.1	7.6	16.6	8843	3165	14,521	1^b^	8.6	8.6	8.7
Egg	Pink	8	89.7	29.9	4049.5	1272	0	7112	2	7.3	5.7	8.9
Sediment		10	10.8	3.6	236.5	24	0	128				

Perylene was not included in TPAH

^a^Cephalothorax

^b^Repeated measure

### Eggs

**Fig. 2:**
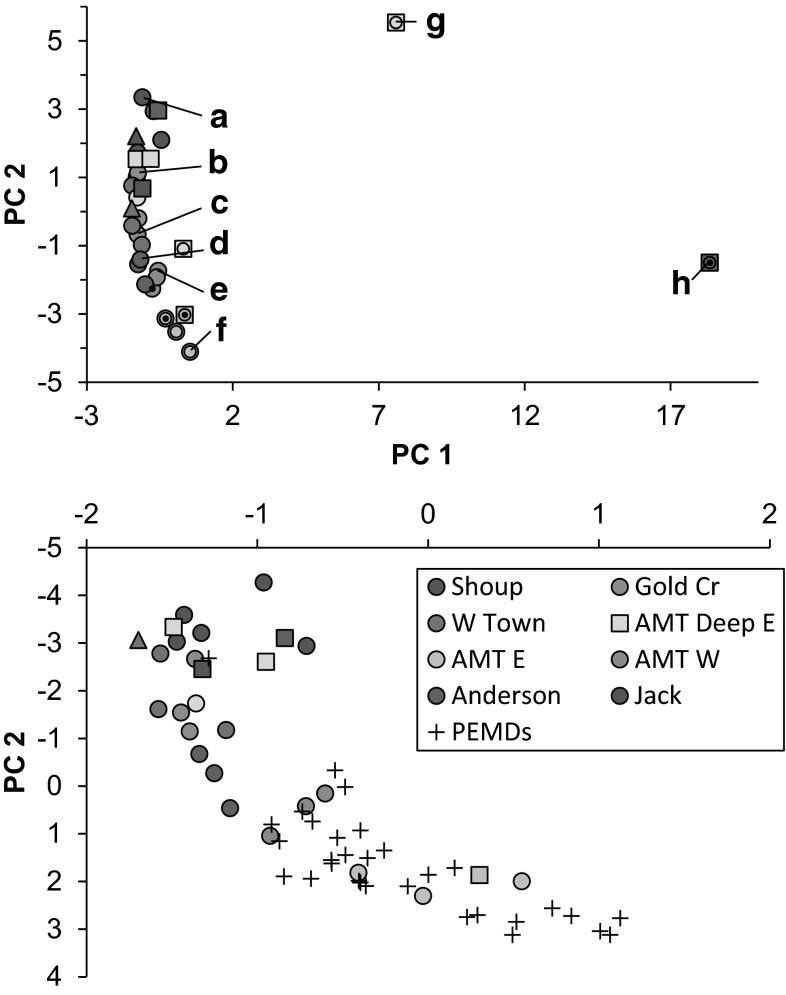
Principal component analysis of shrimp eggs (components 1 vs. 2). *Circles* represent coonstripe shrimp, *triangles* spot shrimp, and *squares* pink shrimp. The *small black dots* identify samples with petrogenic characteristics (based on source modeling) and samples with total PAH concentrations >100 ng/g dry weight are identified with an additional concentric ring. Locations are identified by *color*. *Letters* refer to example chromatograms in Fig. 3. The first two components explained 48 % of the variance. Previous passive sampler (PEMD) data were included in a second PCA analysis (*bottom panel*); restricted scaling does not illustrate the two outliers (f and g at *top*). The PEMDs, which were deployed primarily in the AMT area, were located in the region where oil was detected in eggs. The first two components explained 47 % of the variance in the *bottom graph* (Color figure online)

**Fig. 3:**
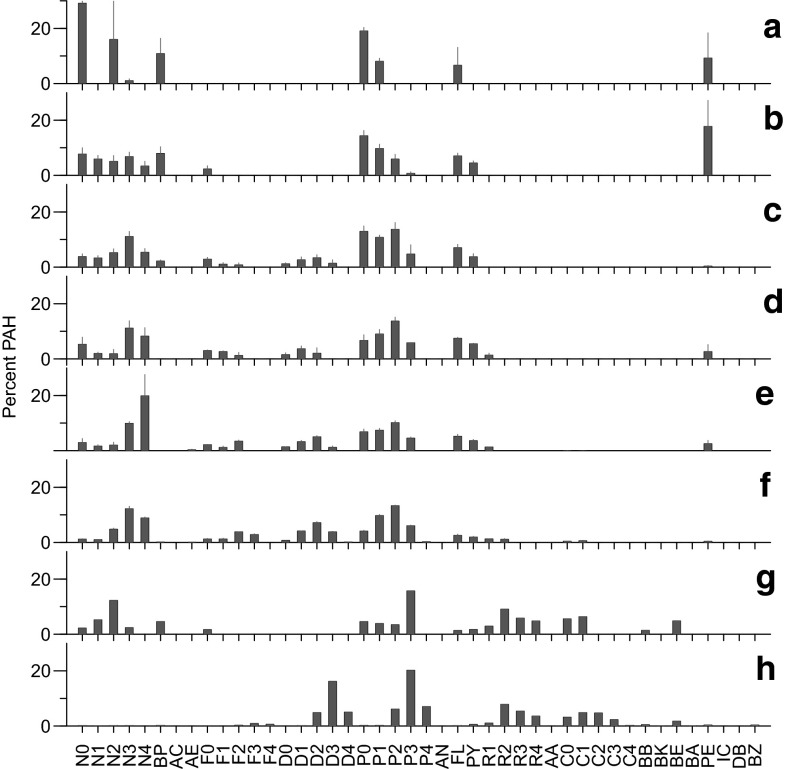
Polynuclear aromatic hydrocarbon composition in shrimp eggs from background conditions to oiled (*a*–*f*) plus an unknown composition (*g*) and weathered oil (*h*). Percentages are mean ± standard error. *Panel letters* correspond to those in Fig. 2

The composition of PAHs in eggs, elucidated by PCA, varied geographically and graded from background conditions to oiled (4 of 32 samples; Fig. 2). Composition in shrimp from Jack Bay and Shoup Bay was consistent with background conditions (Fig. 3a). Composition in eggs changed toward Gold Creek, West Town, and AMT East Deep (Fig. 2); oil influence was not evident in most of these (Fig. 3b–c). Composition in most remaining eggs (Anderson Bay and most AMT sites) was either oiled or was a mixture of oil and background PAHs (Figs. 2, 3e–f). Conditions in eggs from Anderson Bay were the most diverse, ranging from low concentration background PAHs (35–69 ng/g dry weight) to one high-concentration sample (4050 ng/g dry weight) with petrogenic characteristics.

Petrogenic PAHs, determined by composition modeling, were present in coonstripe and pink shrimp eggs at some AMT sites and Anderson Bay (Fig. 3). Oil was detected in pink shrimp eggs at AMT East and in one of two Anderson Bay samples (composition model results were 0.62–0.63; SI 2). Total PAH concentrations in these eggs were the greatest observed, 4065 and 564 ng/g dry weight, respectively. Four additional egg samples had distinct petrogenic fluorene, dibenzothiophene, and phenanthrene composition (SI 2). All samples with oil were collected in the AMT area or Anderson Bay, and they had the highest observed TPAH concentrations (>69 ng/g TPAH) with a single exception, the unusual AMT East Deep sample (121 ng/g, Fig. 3g). The reason for the elevated TPAH concentration in the latter sample is unclear, but it was in the area where oil was observed (AMT) and was weathered more than most (13 % chrysenes). The only other comparably weathered sample was an Anderson Bay sample (15 % chrysenes; Fig. 3h). Perylene dominated the composition in a single Shoup Bay sample (89 %). Insoluble petrogenic alkanes were not observed in egg samples (SI 3), hence the contamination was from a soluble source.

### Muscle

There was no evidence of petrogenic PAHs in shrimp muscle (SI 4). Total PAH concentration varied between 0 and 58 ng/g dry weight with no evidence of contamination (source model results ranged from −0.17 to 0.07). PAHs above MDL included occasional naphthalenes, phenanthrenes, and perylene (SI 4). All TPAH concentrations >10 ng/g were in pink shrimp muscle, and these included all sites with samples (Jack and Anderson Bays and three AMT sites). Total PAH concentrations in muscle were not related to those in corresponding eggs (*r*^2^ = 0.046 for coonstripe shrimp and 0.093 for pink shrimp). Total PAH concentrations in muscle were less than in corresponding eggs in 20 of 21 coonstripe shrimp and all pink shrimp (*n* = 8). Alkanes were rarely detected in muscle (3/41) and were present as a single compound in each instance (typically pristane; SI 3).

### Cephalothoraxes

There was no evidence of petrogenic hydrocarbons in shrimp cephalothoraxes (SI 4). Median TPAH concentration varied among species (4.6, 11.2, and 88.0 ng/g dry weight for coonstripe, spot, and pink shrimp, respectively). There was no evidence of contamination in any samples (model results ranged from −0.18 to 0.22). Naphthalenes and phenanthrenes were the most frequently observed PAHs above MDL; other PAHs were rare (SI 4). Total PAH concentrations in cephalothoraxes were not related to those in corresponding eggs (*r*^2^ = 0.190 for coonstripe shrimp and 0.078 for pink shrimp). Total PAH concentrations in cephalothoraxes were less than in corresponding eggs in all coonstripe shrimp (*n* = 21) and five of eight pink shrimp. Alkanes were frequent in cephalothoraxes (median concentrations were 8401, 12,904, and 30,432 ng/g for coonstripe, spot, and pink shrimp, respectively). Typically detected alkanes were odd or pristane (34/51 samples), thus derived from plants (SI 3). Small quantities of even chain alkanes were observed in the remaining samples, typically at levels <4 % (15 samples). Even alkanes were 9 and 23 % in W Town coonstripe shrimp cephalothoraxes; reasons for this are unclear (SI 5).

### Sediment

Biomarkers provided evidence of oil in the AMT area but petrogenic PAHs were observed only in Jack Bay. The largest observed TPAH concentration was pyrogenic (240 ng/g dry weight) in Shoup Bay. Phytane was present only in the AMT area, corroborating biomarker evidence of oil. Alkane composition was generally consistent with plant production and no UCMs were observed.

**Fig. 4:**
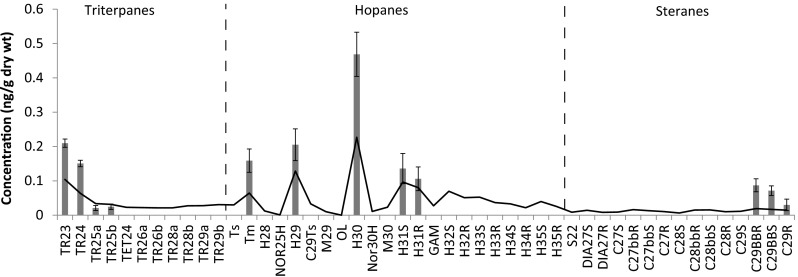
Mean biomarker composition in the Alaska Marine Terminal (AMT) area ± standard error. The *line* indicates composition of Alaska North Slope crude oil. See Table 3 for abbreviations

Biomarkers (triterpanes, hopanes, and steranes) were present at low concentrations only in the AMT area. They were not detected at AMT West or other sites. None were identified by modeling as Alaska North Slope crude oil. However, at the low observed levels, only compounds with the highest concentrations were detected and the patterns present in those compounds were consistent with those in Alaska North Slope crude oil (Fig. 4).

**Fig. 5:**
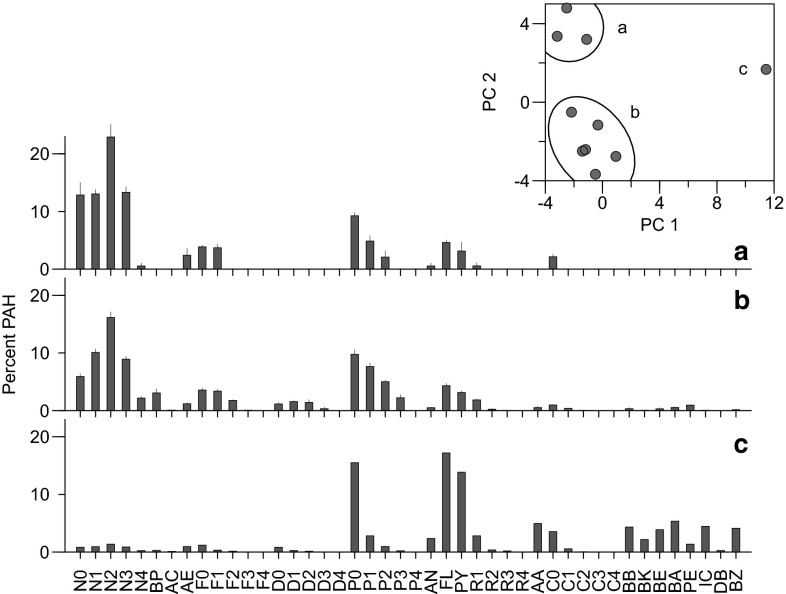
Polynuclear aromatic hydrocarbon composition in benthic sediment and principal component analysis (PCA) of it. The first two PCA components explained 70 % of the data and are illustrated at the *upper right*. *Letters* refer to the composition figures below (mean percentages ± standard error)

PAH composition in sediment varied geographically. PAH composition in sediment formed three groups: West Town, Gold Creek, AMT East Deep formed one group; Jack Bay, Anderson Bay plus the remaining AMT sites formed the second; and Shoup Bay was the third (Fig. 5). Total PAH concentrations were generally low (typically 4–79 ng/g dry weight) and consistent with background conditions (Fig. 5). Composition was petrogenic in Jack Bay (naphthalenes, fluorenes, dibenzothiophenes, and phenanthrenes had petrogenic characteristics), and the TPAH concentration was the second highest (79 ng/g). Total PAH concentration was greatest in Shoup Bay sediment (240 ng/g) and composition was pyrogenic (Fig. 5, SI 6). The higher molecular weight compounds accounted for 26 % of the PAHs, much more than in any other sediment where the range was 0–5 %. Naphthalenes were the dominant homolog in all other sediments.

Few calibrated *n*-alkanes were detected in benthic sediment by GCFID; pristane and phytane were detected by GCMS. Phytane was observed only at AMT sites; it was absent elsewhere, including AMT West. The most likely source of phytane is oil associated with treated ballast water effluent. Small pristane peaks were observed at every site (range 0.6–5.3 ng/g dry), and were likely produced by copepods which synthesized it from chlorophyll-derived phytol (Avigan and Blumer 1968). *n*-C27 was usually present and occasionally *n*-C25 and *n*-C29. These odd-chain alkanes were likely produced by terrestrial plants (Harji et al. 2008; Zhao et al. 2003). Alkane concentrations were typically <100 ng/g dry weight with a single exception at AMT West, 128 ng/g. No UCMs were observed.

## Discussion

Generally low-level petrogenic hydrocarbon contamination was evident in shrimp eggs primarily in the vicinity of the AMT and extending along the southern shore of Port Valdez to Anderson Bay. Shrimp eggs, which are carried externally on female pleopods, are capable of bioaccumulating hydrocarbons from water and sequestering them at detectable levels. An absence of insoluble petrogenic alkanes indicated that hydrocarbons accumulated from a dissolved source. Eggs were the most vulnerable biological compartment to hydrocarbon accumulation; there was no evidence of PAHs from oil in shrimp muscle or cephalothoraxes. Some oil-contaminated sediment was observed in the vicinity of the AMT, coincident with evidence of egg contamination, thus the source of biological contamination was possibly sediment, mediated by aqueous transfer, or contamination of both sediment and eggs was due to direct exposure to aqueous ballast water effluent. Total PAH accumulation was substantially greater in some pink shrimp than in other species, thus differences in habitat utilization (muddy vs. rocky substrate) are potentially important.

Similarities of PAH uptake by shrimp eggs and past observations supports aqueous transfer as the exposure route. Ingestion is not a possible route of contamination in eggs. Maternal transfer was unlikely, because internal PAH concentrations were lower than in eggs and were inconsistent with petrogenic sources, and the hydrocarbon chemistry was consistent with a dissolved source. Insoluble PAHs (those larger than C1-chrysenes) were rarely detected (confined to two samples) and insoluble phytane was not detected in any eggs. Where they are exposed under current conditions (2008–2012), Port Valdez mussels typically acquire only dissolved oil constituents (Payne et al. 2013). Planktonic copepods, which seasonally occupy various depths in the water column, also accumulate dissolved PAHs; insoluble phytane was not detected in them (Carls et al. 2006). Passive samplers (polyethylene membrane devices, PEMDs) deployed in the AMT area in 2001 accumulated dissolved petrogenic constituents (Salazar et al. 2002). PCA comparison of PEMD data with the shrimp egg data reveals that PAH composition in shrimp eggs and in PEMDs in the AMT area was similar and the point clouds overlapped (Fig. 2b). We infer that the hydrocarbon exposure processes observed in 2013 were the same as those active in 2001; in both cases dissolved oil constituents were present in the water and available for accumulation by lipid-rich organisms or surrogate passive samplers. Mean TPAH concentrations in pink shrimp eggs were greater than expected from the historical record but generally comparable for other eggs and all tissues. Concentrations in bay mussels ranged from approximately 7–55 ng/g dry wt. in the past 5 years (2009–2012) (Payne et al. 2013) and approximately 126–296 ng/g in *Neocalanus* copepods collected within Port Valdez (Carls et al. 2006). The median concentration in pink shrimp eggs was 90 ng/g (*n* = 8); loads were substantial in two samples (564 and 4065 ng/g dry wt). Total PAH concentrations in shrimp muscle (Table 5) were below to within the 5-year range observed in mussels.

Total PAH concentrations in sediment in 2013 were lower than those in the past 5 years. For example, TPAH concentrations in sediment ranged from 5 to 15 ng/g dry wt. in the AMT area, below the 24–119 range observed between 2009 and 2012 (Payne et al. 2013). The concentration at Gold Creek (6 ng/g) was less than the previous 15 to 50 ng/g range (Payne et al. 2013) and the 79 ng/g concentration at Jack Bay was less than that in 2004 (2015 ng/g) (Payne et al. 2013).

Petrogenic hydrocarbon accumulation in some shrimp eggs raises concern for embryonic development. In general, PAH quantities in egg tissue were of little concern; TPAH concentrations were typically <100 ng/g dry wt. (24 of 32 samples). However, concentrations in some coonstripe shrimp from the AMT area were in the 200–300 ng/g range (*n* = 3) and TPAH exceeded 100 ng/g in three pink shrimp egg samples in this area (122–564 ng/g). Total PAH was unexpectedly high in one pink shrimp egg sample from Anderson Bay (4065 ng/g), and it had a weathered oil signature. Pacific herring eggs exposed to weathered crude oil were damaged at peak accumulations of approximately 200 ng/g dry wt (Carls et al. 1999), raising concern for potential damage in similarly exposed shrimp embryos. Relationships between exposure timing, incubation time, species differences, and other factors are unknown, making inferences difficult, but it raises the possibility that shrimp development might be impaired by environmental exposures and suggests further study is warranted.

### Risk Assessment for Human Consumption

Shrimp tissue rarely posed risk to human consumers. The PECs in 1 or 2 of 32 shrimp egg samples exceeded the U.S. EPA guidelines but were not significantly elevated in muscle or cephalothorax tissue (*n* = 41 and 51, respectively; Table 6). Exceedances, 3 % for recreational use or 6 % for subsistence use, involved only the two most contaminated egg clutches (pink shrimp from Anderson Bay); compounds larger than chrysenes were culpable (Table 6; Fig. 1). These same two egg samples posed a serious cancer risk to children (ILCR >1.0^−4^). The ILCR exceeded 10^−6^ for children in one additional shrimp egg clutch, but this risk was acceptably low (Table 6). However, the frequency of serious and possible risk was much higher for toddlers (ages 1–2 years) when the U.S. EPA ADAF was included in the risk model (Table 6). Total PAH content in the unusual eggs was 2 to >3 standard deviations above the mean. This contamination was likely the result of recreational or commercial boating activity not associated with the AMT, because they were collected in Anderson Bay, well removed from the terminal. Consistent with this inference, Anderson Bay is frequently utilized as an anchorage by boats. There were no indications of unrelated contamination events during the field collection or in the laboratory. The conclusion that Port Valdez shrimp generally pose little cancer risk for people older than age 2 years is consistent with a study completed 1 year after the *Exxon Valdez* oil spill, which estimated that the risk of cancer was low for people consuming fish harvested from the spill area (Bolger et al. 1996).

**Table 6:** Frequency of samples exceeding various potency equivalent concentrations (PEC) and incremental lifetime cancer risk (ILCR) levels

Tissue	*n*_total_	Potency		Serious risk (%)		Possible risk (%)		Acceptable risk (%)	
		PEC_subsist_	PEC_rec_	ILCR_adult_	ILCR_child_	ILCR_adult_	ILCR_child_	ILCR_adult_	ILCR_child_
Eggs	32	6.3	3.1	0.0	3.1	3.1	6.3	6.3	9.4
Muscle	41	0.0	0.0	0.0	0.0	0.0	0.0	0.0	0.0
c-Thorax^a^	51	0.0	0.0	0.0	0.0	0.0	0.0	0.0	0.0
EPA adjusted estimates, age 2 years to adult									
Eggs	32				3.1		6.3		15.6
Muscle	41				0.0		0.0		0.0
c-Thorax^a^	51				0.0		0.0		0.0
EPA adjusted estimates, age 1 year to adult									
Eggs	32				15.6		90.6		100.0
Muscle	41				0.0		2.4		29.3
c-Thorax^a^	51				0.0		2.0		51.0

PEC exceedance criteria were 0.67 ng/g ww for subsistence use (PEC_subsist_) and 5.47 for recreational use (PEC_rec_). ILCR of 10^−5^ to 10^−6^ is considered acceptable (CCME 2008; USEPA 2000; Ylitalo et al. 2012); values exceeding 10^−6^ are labeled acceptable, >10^−5^ are labeled possible, and >10^−4^ are labeled serious. Estimates were completed independently for adults (ILCR_adult_) and children (ILCR_child_). Samples where ILCR ≤10^−6^ are not included in this summary

^a^Cephalothorax

## Conclusions

Hydrocarbon tainting of shrimp muscle is not a concern for the shrimp fishery in Port Valdez and observed concentrations generally do not pose a human health risk. PAH accumulation in some shrimp eggs potentially may impair embryo development, but PAH distributions among shrimp and composition within eggs suggests that human health risk is generally unlikely except for children younger than age 2 years. Petrogenic contamination was not observed in shrimp muscle and cephalothoraxes. Differences in shrimp habitat utilization may be important; pink shrimp eggs accumulated more PAHs than coonstripe shrimp eggs despite similarities in lipid content. In general, PAH loads were consistent with local time series data in other species and with uptake from water; PAH composition was consistent with passive sampler observations completed more than a decade earlier. Hydrocarbon levels in the majority of samples were not concerning. Most elevated concentrations occurred in the AMT area, but the egg sample with the greatest contamination was observed in Anderson Bay—approximately 17 km west of the ballast water treatment facility. Thus, unrelated boat activity is suspected.

## Electronic supplementary material

Below is the link to the electronic supplementary material.

Supplementary material 1 (DOCX 301 kb)

## Footnotes

1 Reference to trade names does not imply endorsement by the National Marine Fisheries Service, NOAA.
