# Supplementary material for: Polynuclear Aromatic Hydrocarbons in Port Valdez Shrimp and Sediment
Source: Arch Environ Contam Toxicol. 2016 Mar 31;71:48–59. doi: 10.1007/s00244-016-0279-3 (PMC4906058; doi:10.1007/s00244-016-0279-3)
Supplement: Supplementary file 1 — Supplementary material 1 (DOCX 301 kb) [file 244_2016_279_MOESM1_ESM.docx]

**Supporting information**

**SI 1**. Mean shrimp carapace lengths (± sd) by study site (top) and relationship between carapace length and egg mass (bottom). Shrimp species are coonstripe (coon), pink, and spot.


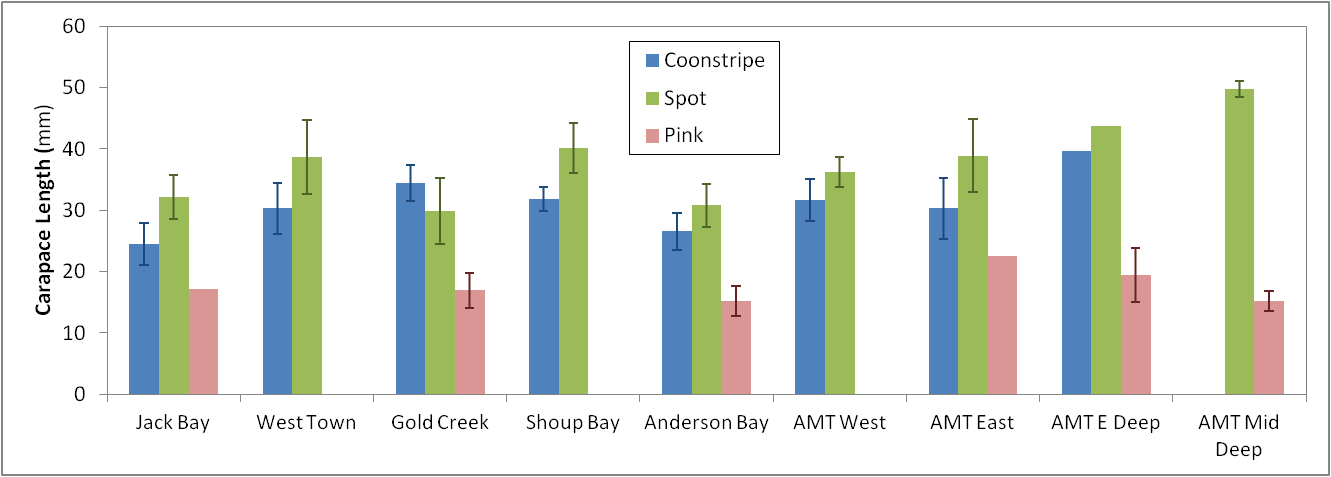


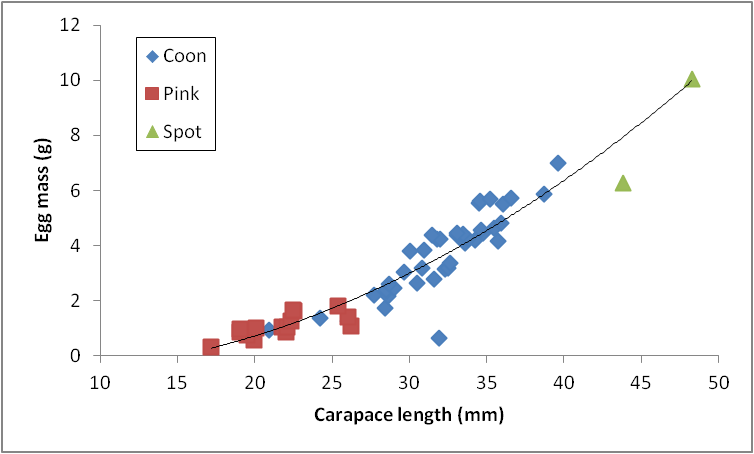


**SI 2**. PAHs in shrimp eggs. SIN is sample identification number, TPAH is total polynuclear aromatic hydrocarbon concentration, pery is perylene, model results are based on updated version of Carls (2006), cAlk is total calibrated *n*-alkanes, oiled? presents conclusions about the samples (no = not oiled, ? = oil plausible, oil = soluble oil constituents present, or unknown, pNaph is proportion of naphthalenes (range 0.0 to 1.0, of TPAH), and proportions of fluorenes (pFluo), dibenzothiophenes (pDiben), chrysenes (pChrys), phenanthrenes (pPhen), fluoranthene-pyrenes (pFP), and other higher molecular weight compounds (pHMW).

| **SIN** | **Date** | **Location** | **Species** | **TPAH ng/g dry** | **TPAH-pery ng/g dry** | **TPAH model** | **cAlk ng/g dry** | **Oiled?** | **pNaph** | **pFluo** | **pDiben** | **pChrys** | **pPhen** | **pFP** | **pHMW** |
| --- | --- | --- | --- | --- | --- | --- | --- | --- | --- | --- | --- | --- | --- | --- | --- |
| 20130201 | 4/9/2013 | Jack Bay | coonstripe | 16 | 16 | 0.12 | 2378 | no | 0.35 | 0.00 | 0.00 | 0.00 | 0.44 | 0.21 | 0.00 |
| 20130202 | 4/9/2013 | Jack Bay | coonstripe | 26 | 26 | 0.05 | 0 | no | 0.72 | 0.00 | 0.00 | 0.00 | 0.28 | 0.00 | 0.00 |
| 20130203 | 4/9/2013 | Jack Bay | coonstripe | 5 | 5 | 0.10 | 1450 | no | 0.00 | 0.00 | 0.00 | 0.00 | 0.52 | 0.48 | 0.00 |
| 20130206 | 4/9/2013 | West Town | coonstripe | 30 | 30 | -0.15 | 556 | no | 0.36 | 0.00 | 0.08 | 0.00 | 0.40 | 0.15 | 0.00 |
| 20130207 | 4/9/2013 | West Town | coonstripe | 52 | 52 | 0.18 | 860 | no | 0.30 | 0.11 | 0.03 | 0.00 | 0.41 | 0.15 | 0.00 |
| 20130208 | 4/9/2013 | West Town | coonstripe | 40 | 40 | -0.10 | 1996 | no | 0.24 | 0.00 | 0.00 | 0.00 | 0.53 | 0.23 | 0.00 |
| 20130209 | 4/9/2013 | Gold Creek | coonstripe | 51 | 51 | 0.20 | 785 | no | 0.28 | 0.07 | 0.10 | 0.00 | 0.40 | 0.16 | 0.00 |
| 20130210 | 4/9/2013 | Gold Creek | coonstripe | 37 | 37 | -0.17 | 1441 | no | 0.52 | 0.00 | 0.00 | 0.00 | 0.35 | 0.13 | 0.00 |
| 20130211 | 4/9/2013 | Gold Creek | coonstripe | 49 | 49 | 0.07 | 1375 | no | 0.41 | 0.04 | 0.09 | 0.00 | 0.38 | 0.08 | 0.00 |
| 20130213 | 4/9/2013 | Shoup Bay | coonstripe | 129 | 15 | -0.08 | 1098 | no | 0.02 | 0.00 | 0.00 | 0.00 | 0.05 | 0.04 | 0.89 |
| 20130214 | 4/9/2013 | Shoup Bay | coonstripe | 33 | 33 | -0.20 | 22833 | no | 0.36 | 0.00 | 0.00 | 0.00 | 0.45 | 0.19 | 0.00 |
| 20130215 | 4/9/2013 | Shoup Bay | coonstripe | 35 | 35 | -0.10 | 656 | no | 0.36 | 0.05 | 0.00 | 0.00 | 0.41 | 0.18 | 0.00 |
| 20130217 | 4/9/2013 | Anderson | coonstripe | 58 | 58 | -0.02 | 733 | no | 0.36 | 0.04 | 0.06 | 0.00 | 0.38 | 0.17 | 0.00 |
| 20130218 | 4/9/2013 | Anderson | coonstripe | 69 | 69 | 0.43 | 969 | ? | 0.34 | 0.05 | 0.13 | 0.00 | 0.36 | 0.12 | 0.00 |
| 20130219 | 4/9/2013 | Anderson | coonstripe | 43 | 43 | 0.07 | 380 | no | 0.28 | 0.00 | 0.11 | 0.00 | 0.45 | 0.16 | 0.00 |
| 20130221 | 4/9/2013 | AMT W | coonstripe | 96 | 96 | 0.50 | 755 | oil | 0.50 | 0.07 | 0.10 | 0.00 | 0.25 | 0.08 | 0.00 |
| 20130222 | 4/9/2013 | AMT W | coonstripe | 74 | 74 | 0.43 | 985 | ? | 0.27 | 0.10 | 0.09 | 0.00 | 0.39 | 0.15 | 0.00 |
| 20130223 | 4/9/2013 | AMT W | coonstripe | 71 | 71 | 0.43 | 996 | ? | 0.30 | 0.07 | 0.14 | 0.00 | 0.36 | 0.13 | 0.00 |
| 20130225 | 4/9/2013 | AMT E | coonstripe | 270 | 270 | 0.40 | 347 | oil | 0.30 | 0.09 | 0.18 | 0.01 | 0.35 | 0.08 | 0.00 |
| 20130226 | 4/9/2013 | AMT E | coonstripe | 120 | 120 | 0.50 | 1764 | oil | 0.31 | 0.10 | 0.16 | 0.00 | 0.35 | 0.08 | 0.00 |
| 20130227 | 4/9/2013 | AMT E | coonstripe | 193 | 193 | 0.33 | 637 | oil | 0.27 | 0.10 | 0.19 | 0.01 | 0.35 | 0.09 | 0.00 |
| 20130229 | 4/9/2013 | AMT E deep | coonstripe | 22 | 22 | 0.17 | 895 | no | 0.60 | 0.00 | 0.00 | 0.00 | 0.28 | 0.12 | 0.00 |
| 20130234 | 4/9/2013 | W Town | spot | 17 | 17 | -0.08 | 3165 | no | 0.36 | 0.10 | 0.06 | 0.00 | 0.32 | 0.09 | 0.00 |
| 20130242 | 4/9/2013 | Shoup Bay | spot | 8 | 8 | -0.03 | 14521 | no | 0.27 | 0.12 | 0.00 | 0.00 | 0.42 | 0.19 | 0.00 |
| 20130259 | 4/10/2013 | Jack Bay | pink | 58 | 58 | -0.02 | 2279 | no | 0.45 | 0.00 | 0.00 | 0.00 | 0.26 | 0.28 | 0.00 |
| 20130264 | 4/10/2013 | Anderson | pink | 35 | 35 | 0.15 | 0 | no | 0.80 | 0.00 | 0.00 | 0.00 | 0.20 | 0.00 | 0.00 |
| 20130265 | 4/10/2013 | Anderson | pink | 4065 | 4050 | 0.62 | 7112 | oil | 0.01 | 0.02 | 0.26 | 0.15 | 0.34 | 0.19 | 0.03 |
| 20130268 | 4/10/2013 | AMT E | pink | 564 | 564 | 0.63 | 834 | oil | 0.30 | 0.10 | 0.16 | 0.02 | 0.37 | 0.05 | 0.00 |
| 20130269 | 4/10/2013 | AMT E deep | pink | 119 | 119 | 0.25 | 1607 | ? | 0.13 | 0.03 | 0.15 | 0.00 | 0.65 | 0.04 | 0.00 |
| 20130270 | 4/10/2013 | AMT E deep | pink | 121 | 121 | 0.37 | 936 | unknown | 0.22 | 0.00 | 0.00 | 0.13 | 0.31 | 0.27 | 0.07 |
| 20130271 | 4/10/2013 | AMT E deep | pink | 30 | 30 | 0.32 | 874 | no | 0.34 | 0.00 | 0.00 | 0.00 | 0.52 | 0.14 | 0.00 |
| 20130272 | 4/10/2013 | AMT E deep | pink | 61 | 61 | 0.28 | 2471 | no | 0.46 | 0.00 | 0.00 | 0.00 | 0.49 | 0.05 | 0.00 |

**SI 3.**  Mean normalized alkane composition in tissue.


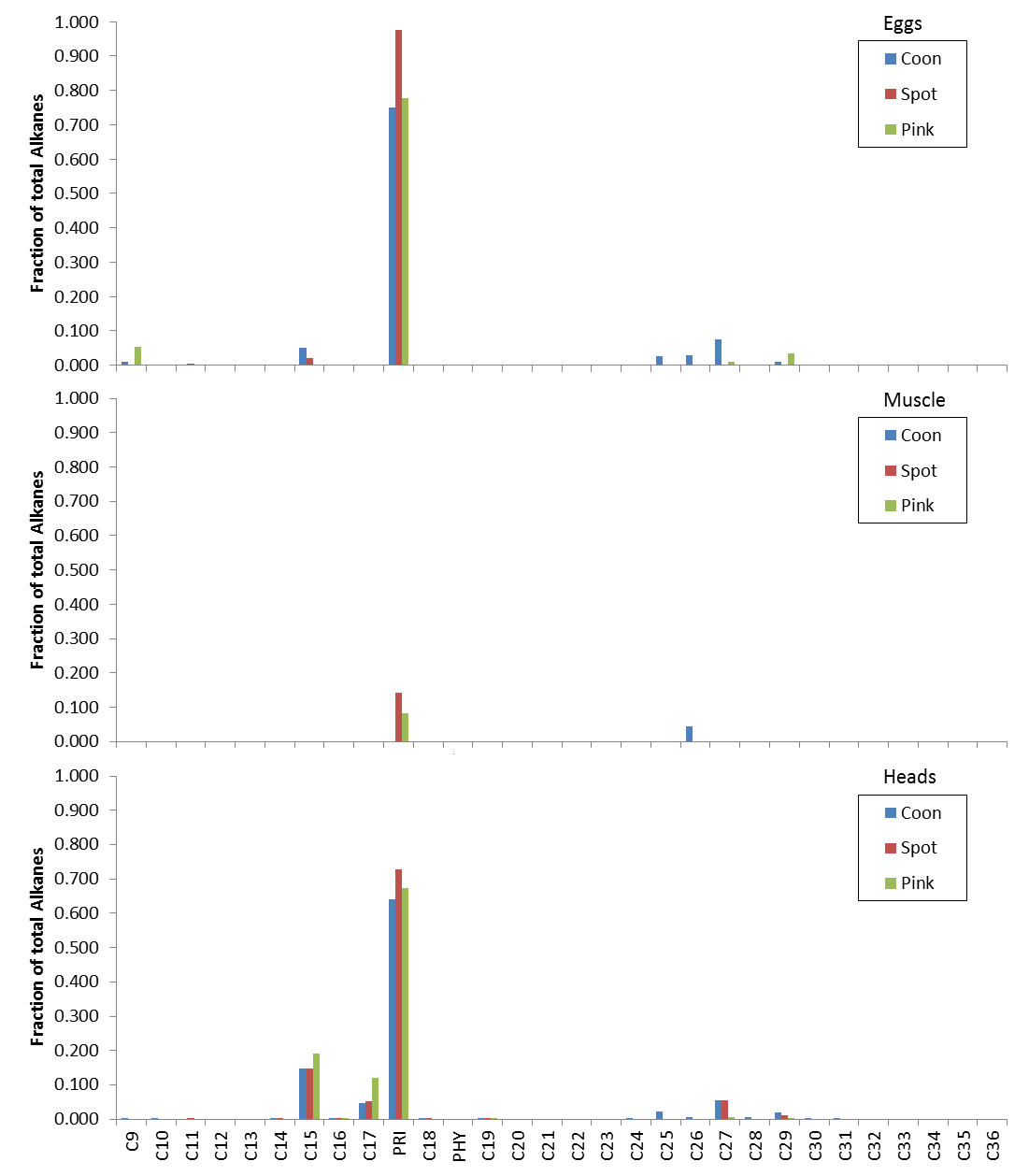


**SI 4.** Mean normalized TPAH concentration in shrimp muscle and heads. See Appendix B for abbreviations.


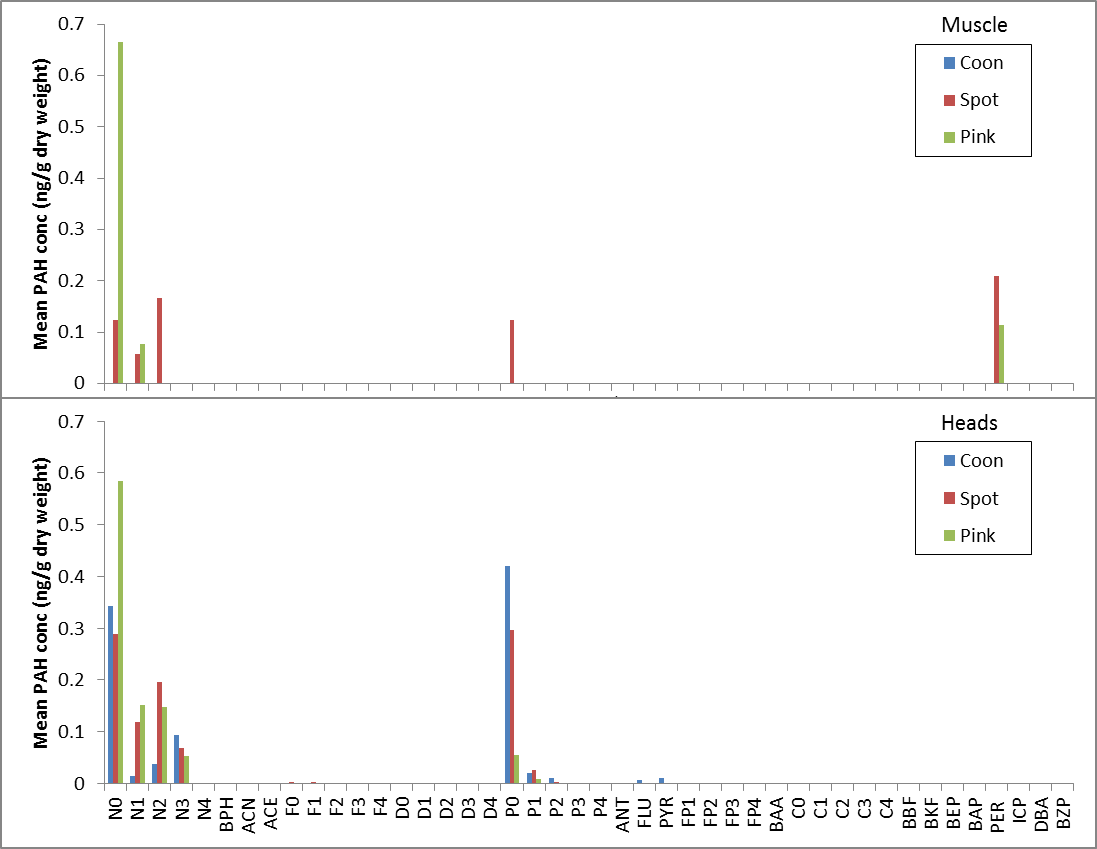


**SI 5.** Alkanes in shrimp cephalothoraxes, W. Town. Composition in the top panel is representative of most alkanes in shrimp heads, with only odd chains present or a dominance of odd chains. The middle and bottom panels are two unusual samples (coonstripe shrimp) where even alkanes were present. Pink shrimp were not captured at this site.

**SI 6**. Hydrocarbons in benthic sediment. Concentrations are ng/g dry weight. TPAH = total polynuclear aromatic hydrocarbons, pS3 = PAH source model result (unitless, range -1.0 to +1.0), cAlk = total calibrated *n*-alkanes, TRI = tricyclic terpanes, HOP = hopanes, and STE = steranes.

| **SIN** | **Date** | **Location** | **TPAH** | **pS3** | **cAlk** | **TRI** | **HOP** | **STE** |
| --- | --- | --- | --- | --- | --- | --- | --- | --- |
| 20130116 | 2/26/2013 | Shoup Bay | 239.8 | -0.67 | 0.0 | 0.000 | 0.000 | 0.000 |
| 20130111 | 2/26/2013 | Gold Creek | 6.5 | -0.03 | 0.8 | 0.000 | 0.000 | 0.000 |
| 20130105 | 2/26/2013 | West Town | 3.6 | 0.03 | 1.0 | 0.000 | 0.000 | 0.000 |
| 20130143 | 2/27/2013 | AMT East Deep | 4.6 | 0.07 | 0.8 | 0.007 | 0.011 | 0.004 |
| 20130145 | 2/27/2013 | AMT East | 9.9 | -0.03 | 0.6 | 0.011 | 0.042 | 0.008 |
| 20130135 | 2/27/2013 | AMT Mid Deep | 14.7 | -0.03 | 0.6 | 0.006 | 0.018 | 0.003 |
| 20130137 | 2/27/2013 | AMT West Deep | 10.0 | 0.10 | 0.7 | 0.006 | 0.008 | 0.000 |
| 20130130 | 2/26/2013 | AMT West | 11.7 | -0.03 | 0.9 | 0.000 | 0.000 | 0.000 |
| 20130124 | 2/26/2013 | Anderson | 30.4 | -0.18 | 0.5 | 0.000 | 0.000 | 0.000 |
| 20130118 | 2/26/2013 | Jack Bay | 79.1 | 0.33 | 0.2 | 0.000 | 0.000 | 0.000 |
